# Effect of Web-Based Messages on Girls’ Knowledge and Risk Perceptions Related to Cigarette Smoke and Breast Cancer: 6-Month Follow-Up of a Randomized Controlled Trial

**DOI:** 10.2196/resprot.3282

**Published:** 2014-09-30

**Authors:** Jennifer Schwartz, Joan L Bottorff, Pamela A Ratner, Carolyn Gotay, Kenneth C Johnson, Jasmina Memetovic, Chris G Richardson

**Affiliations:** ^1^Yale UniversitySchool of MedicineCenter for Outcomes Research & Evaluation (CORE)New Haven, CTUnited States; ^2^University of British ColumbiaSchool of NursingUniversity of British ColumbiaKelowna, BCCanada; ^3^University of British ColumbiaSchool of NursingUniversity of British ColumbiaVancouver, BCCanada; ^4^University of British ColumbiaSchool of Population & Public HealthUniversity of British ColumbiaVancouver, BCCanada; ^5^University of OttawaDepartment of Epidemiology and Community MedicineUniversity of OttawaOttawa, ONCanada

**Keywords:** secondhand smoke, breast cancer, Web-based health promotion, adolescents

## Abstract

**Background:**

Evidence indicating an association between cigarette smoke exposure and an increase in breast cancer risk highlights the need for health messages that aim to prevent smoking initiation and reduce secondhand smoke (SHS) exposure among adolescent girls.

**Objective:**

This study aimed to evaluate the efficacy of targeted gender-sensitive, breast cancer-specific, Web-based messages about the increased risk of breast cancer associated with cigarette smoke exposure. Outcomes assessed 6 months postmessage delivery included nonsmoking adolescent girls’ knowledge of the link between cigarette smoke exposure and breast cancer, perceptions of breast cancer risk associated with cigarette smoke, smoking behavior and intentions, and stage of change related to avoidance of secondhand smoke.

**Methods:**

A prospective randomized controlled trial was used to compare standard (control) messages with targeted gender- and Aboriginal status-sensitive, breast cancer-specific (intervention) messages. Messages were delivered online to 618 nonsmoking girls, aged 13 to 15 years, clustered in 74 Canadian secondary schools.

**Results:**

Compared with the control group, girls in the intervention group were significantly more likely to report that breast cancer is an illness caused by cigarette smoke (adjusted relative risk [ARR] 1.33, 95% CI 1.05-1.68) and to agree that exposure to SHS increases their risk of breast cancer (ARR 1.10, 95% CI 1.02-1.20). No significant effects were observed for a change in smoking status, intention to try smoking, or stage of change related to avoidance of SHS.

**Conclusions:**

Compared with standard messages, targeted gender-sensitive, breast cancer-specific messages had a stronger influence on girls’ knowledge and perceived risk of cigarette smoke exposure as a risk factor for breast cancer. Brief information-based interventions delivered over the Internet have the potential to provide effective health promotion that could be broadly disseminated and lead to long-term effects on girls’ knowledge and risk perceptions about cigarette exposure and breast cancer.

## Introduction

Recent evidence indicates that cigarette smoking and secondhand smoke (SHS) exposure are associated with an increase in premenopausal breast cancer risk [1-7]. In 2009, the Canadian Expert Panel on Tobacco Smoke and Breast Cancer concluded that, based on epidemiologic and toxicological studies, the associations between cigarette smoking and breast cancer, as well as between long-term regular SHS exposure and premenopausal breast cancer “are consistent with causality” [2-8]. Since the Canadian report, 2 large cohort studies—the Nurses’ Health Study that examined 8772 breast cancer cases [7] and a Norwegian cohort study examining 7490 cases [9]—clearly demonstrated that the critical window of exposure is from menarche to first childbirth, and confirmed what Ha et al had discovered in a smaller US cohort study in 2007 [10]. In all 3 of these cohort studies a clear dose-response relationship was evident—the longer females smoked between menarche and first childbirth, and the more they smoked during that time period, the greater their risk for breast cancer. Additionally, smoking after first childbirth did not increase risk in these studies [7,9]. More recently, analyses from 2 large cohort studies with lifetime assessments of SHS indicated increased breast cancer risk associated with high lifetime SHS exposure [3,11].

The increased risk of premenopausal breast cancer associated with smoking and SHS, especially exposure between menarche and first childbirth, has direct implications for breast cancer prevention strategies. In a recent Canadian survey, 10% of youth in grades 10-12 self-identified as current smokers [12]. The prevalence of youth who had ever tried smoking a cigarette was 16% in grades 6-9 and 40% in grades 10-12, with the average age of tobacco initiation among Canadian girls in 2011 being 13 years [12]. Further, an estimated 22% of youth in grades 5-12 were exposed daily or almost daily to SHS in their homes [12,13]. Although research identifying cigarette exposure as a modifiable risk factor for breast cancer was published in 2009, few interventions have aimed to increase awareness of the causal link between cigarette smoke and increased risk of breast cancer in premenopausal women [14].

From a cancer prevention perspective, targeting interventions toward adolescent girls is particularly important because it is during periods of breast development that cigarette smoke exposure appears to increase the risk of breast cancer. The potential effectiveness of targeting tobacco control messages that highlight the benefits of reducing breast cancer risk toward adolescent girls is also supported by health behavior theory. The pubertal period is marked by heightened awareness of physical (ie, breast development) and psychological (ie, gendered social identity) changes, and can therefore be exploited as a teachable moment for breast cancer prevention [15]. As such, targeted messages linking breast cancer risk with tobacco exposure may hold distinct advantages over general messages about smoking and cancer that youth may dismiss because they perceive the risk to be associated with a distant consequence that is not relevant to their immediate health [16]. In designing breast cancer-specific messages targeting adolescent girls that address the risk of tobacco exposure, it has been recommended that interventions link tobacco exposure to breast cancer in ways that are gender-sensitive, in that messages are relevant and appropriate for girls in this age group (eg, avoiding sexualized images of breasts), reflect the context of tobacco use within youths’ social world, and be attuned to gender-related issues (eg, emerging femininities and girls’ peer relations) [14].

Several meta-analyses and systematic reviews indicate that targeted messages are more persuasive than generic messages, and lead to greater improvements in outcomes including behavioral intentions, behavior change, and attitudes [17-22]. The potential benefit of using targeted gender-sensitive messaging in the context of a teachable moment is also supported by the results of a qualitative study recently conducted by our group. The findings of this study indicated that adolescents seemed to be more receptive to gender-sensitive messages about the relationship between cigarette smoke and breast cancer than they were to standard or putatively gender-neutral messages [23].

The rapid expansion in adolescents’ use of the Internet has led to the characterization of the Internet as an ideal channel for risk factor screening (eg, cigarette smoke exposure and/or smoking behavior) that can be coupled with the delivery of targeted health promotion interventions to reduce cigarette smoke exposure [24]. Digital technology-based interventions can be rapidly disseminated, and can include interactive components that engage youth [25]. Recent studies also indicate that youth-friendly, socially-oriented, and Web-based health messages and interventions can positively affect adolescents’ smoking behavior [17,26,27]. Web-based interventions that deliver gender-sensitive, health-related information on the relationship between cigarette smoke and breast cancer thus appear to represent a promising means of reducing adolescent girls’ exposure to cigarette smoke.

Although Web-based interventions for adolescents have great potential, recent investigations of Web-based smoking cessation interventions for adolescents have not yet demonstrated substantial gains in efficacy [26,28-30]. However, interventions that aimed to prevent cigarette smoking initiation among nonsmokers have been more successful [17,31,32]. Given the limited success associated with Web-based smoking cessation interventions, it may be more productive to focus on the development of Web-based interventions that seek to prevent smoking initiation and to reduce SHS exposure among nonsmoking adolescent girls.

The aim of this study was to investigate the efficacy of targeted gender- and Aboriginal status-sensitive, breast cancer-specific, Web-based messages focused on the increased risk of breast cancer associated with cigarette smoke exposure. The outcomes included nonsmoking adolescent girls’ (1) knowledge of the causal link between cigarette smoke exposure and breast cancer, (2) perceptions of breast cancer risk associated with exposure to cigarette smoke, (3) smoking initiation, (4) intentions to smoke in the future, and (5) stage of change related to avoidance of SHS at follow-up (6 months following the message delivery). We hypothesized that compared with a control group that received a standard message describing the carcinogenic effects of tobacco smoke, a greater proportion of girls exposed to the targeted, disease-specific intervention would identify exposure to cigarette smoke as a cause of, and risk factor for, breast cancer. We also hypothesized that compared with girls who received a standard message, a smaller proportion of girls who received the targeted, disease-specific intervention would try smoking, report intentions to try smoking in the future, and report doing nothing to avoid SHS exposure.

## Methods

### Overview

The study described in this article, entitled Supporting Tailored Approaches to Reducing Tobacco (START): Decreasing Breast Cancer Incidence, was a cluster randomized controlled trial nested within an ongoing Web-based prospective cohort study, the British Columbia Adolescent Substance Use Survey (BASUS). The BASUS study began enrolling students from 48 participating secondary schools in the fall of 2009 and surveyed participants every 6 months until the fall of 2012. All BASUS participants were 13 years of age or older, able to read and complete a Web-based survey in English, and provided informed consent, as well as written parental consent in schools where required. Although the majority of students completed their surveys online outside of school time, others completed the survey in school computer labs during scheduled class time. Students received reminders to complete each wave of the BASUS survey via school-based posters and announcements, as well as via personal email if requested by the participant. Students could retrieve their passwords via email using a lost password button on the BASUS website. All participants received a $25 gift card as an honorarium for each wave of the survey completed, and the response rates for individual schools ranged from 2% to 100%, with a 20% average. In the spring of 2011, schools were stratified into groups containing a similar number of study participants and randomly assigned to have their students receive either the targeted, breast cancer-specific intervention or the control message. The follow-up assessment was completed approximately 6 months later as part of the next wave of the BASUS study. For a complete description of the design of the randomized controlled trial and CONSORT statement, please refer to Richardson et al [33]. Ethical approval for both the BASUS and START studies was obtained from the University of British Columbia’s Behavioural Research Ethics Board.

### Intervention Condition

**Figure 1:**
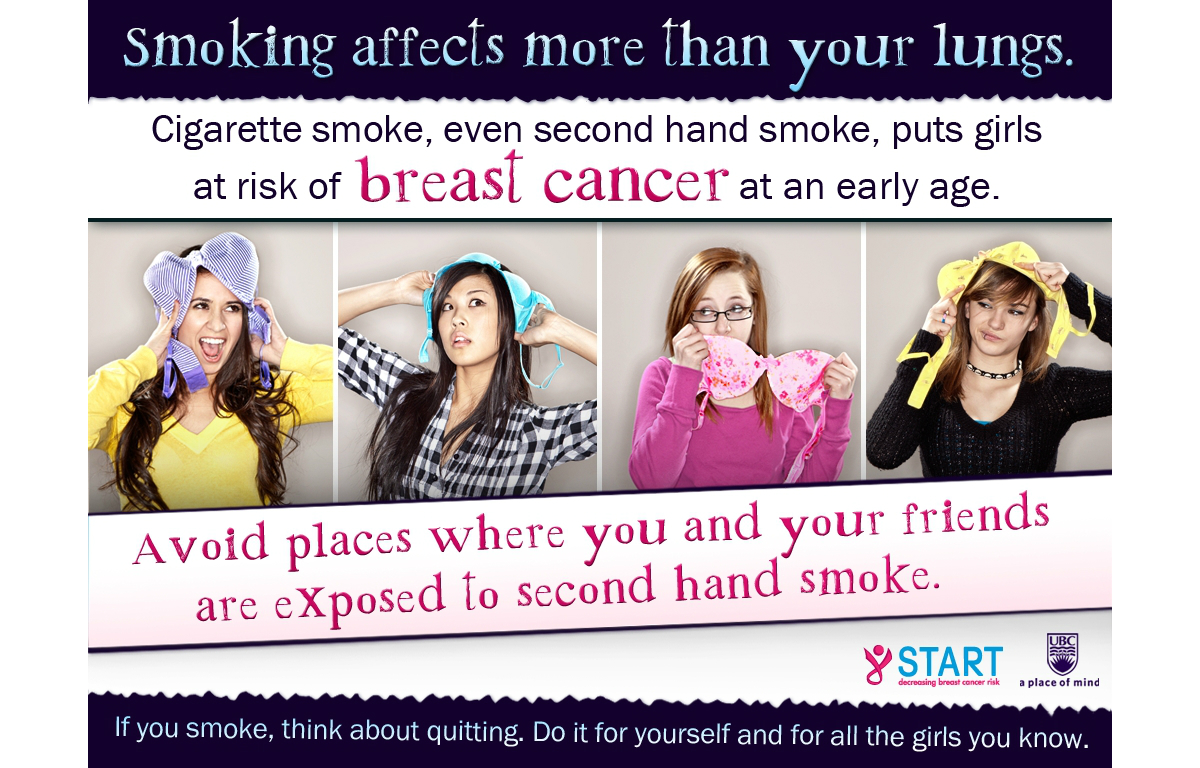
Gender-targeted intervention message for girls. Source: Created by START study authors, who hold copyright to the image.

**Figure 2:**
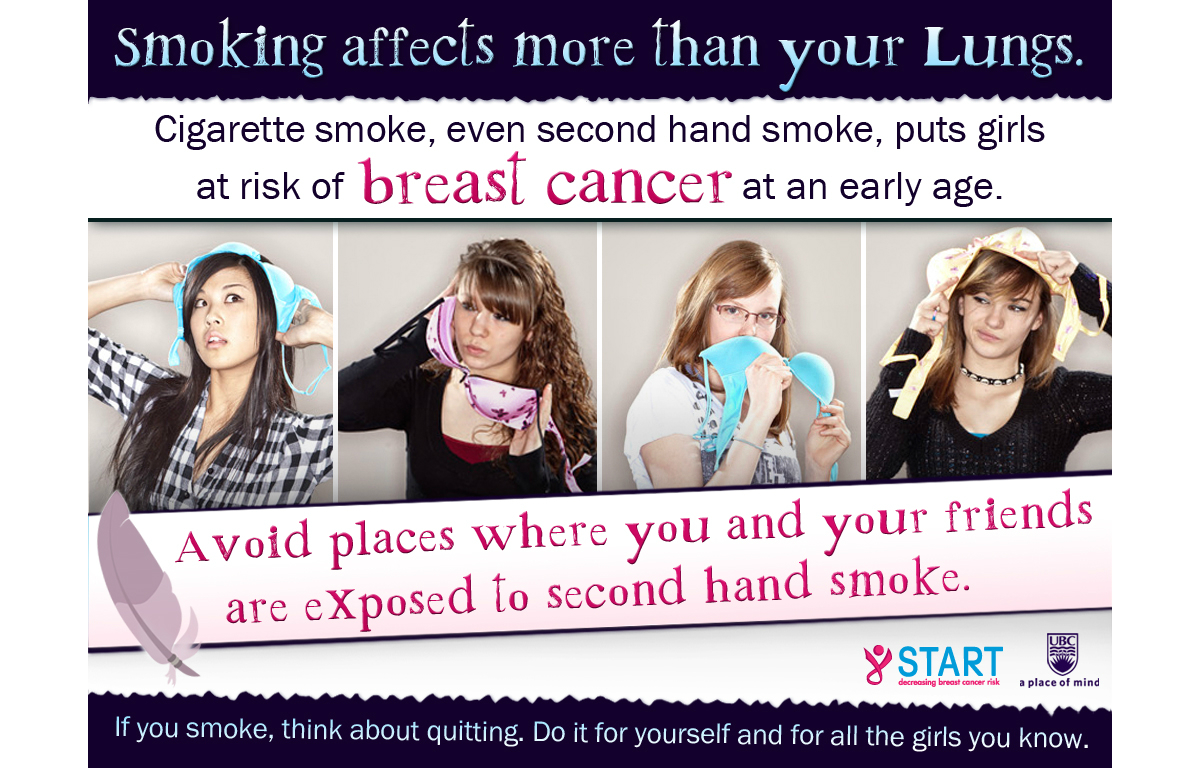
Gender- and Aboriginal status-sensitive, targeted intervention message for girls. Source: Created by START study authors, who hold copyright to the image.

A tailored intervention message was designed to be sensitive to gender and Aboriginal status [33]. The resulting message included images of four different girls playfully holding bras, with the statement “Smoking affects more than your lungs” followed by “Cigarette smoke, even secondhand smoke, puts girls at risk for breast cancer at an early age.” The message also included the following suggestions for action: “Avoid places where you and your friends are exposed to secondhand smoke. If you smoke, think about quitting. Do it for yourself and all the girls you know.” An example of the intervention message is displayed in Figure 1. For girls self-identifying as Aboriginal, the same message was received at baseline, with the addition of a feather watermark (displayed in Figure 2), an important ceremonial symbol among people of First Nation or Aboriginal ancestry [33].

Following receipt of the intervention message at baseline, adolescent girls answered the following yes/no question: “Would you be interested in receiving information about the connection between cigarette smoke and breast cancer?” If a participant responded “Yes,” she received additional information about the risk of cigarette smoke exposure and breast cancer upon completion of the survey (available on request).

### Control Condition

**Figure 3:**
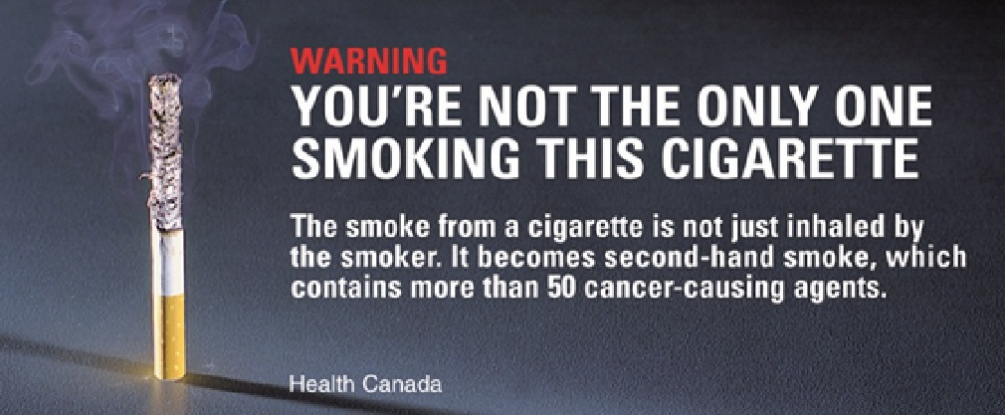
START control message. Source: Health Canada.

Students in the control group were presented with a standard message that cigarette smoke contains carcinogenic agents. The message was sourced from Health Canada’s online library of health labels and warnings for cigarette tobacco [34]. This message included an image of an ash-laden burning cigarette standing against a black background (Figure 3), with the following statement:

Warning, you’re not the only one smoking this cigarette. The smoke from a cigarette is not just inhaled by the smoker. It becomes secondhand smoke, which contains more than 50 cancer-causing agents

Following receipt of the standard message at baseline, adolescent girls answered the following yes/no question: “Would you be interested in receiving information about the connection between cigarette smoke and cancer?” If a participant responded “Yes,” she received additional general information about the risk of cigarette smoke exposure and cancer upon completion of the survey questions (available on request).

### Participants

Participants included in the current analysis were nonsmoking adolescent girls, aged 13 to 15 years, who participated in the BASUS study. For baseline characteristics postrandomization, please refer to Richardson et al [33].

### Measures

The baseline data collection for the current study occurred between April and June 2011; the follow-up data were collected between October and December 2011. The baseline survey questions determined the participants’ demographic and SHS exposure characteristics, including age, ethnicity, family income (below average, average, or above average), and family history of breast cancer (yes or no). In addition, smoking behavior (ever tried smoking), intentions to smoke in the future, SHS exposure (parental smoking status, peer smoking status, cigarette smoke exposure at home, and extent of past month’s exposure to SHS), and interest in receiving more information were assessed at baseline.

Approximately 6 months after the baseline dissemination of the intervention and control messages, the subsequent wave of the BASUS survey was administered. The following question was used to assess the girls’ knowledge of the connection between cigarette smoke exposure and breast cancer: “Which of the following illnesses have been shown to be caused by exposure to cigarette smoke?”, with the following response options: AIDS, arthritis, asthma, bladder cancer, breast cancer, common cold, diabetes, heart disease, lung cancer, measles, schizophrenia, stomach cancer, or none of the above. Risk perceptions regarding cigarette smoke exposure and breast cancer were assessed by asking participants to respond to the following statement: “Being exposed to secondhand cigarette smoke increases my risk of getting breast cancer”, with the following response options: strongly agree, agree, disagree, or strongly disagree. Intentions to smoke were measured with the following question: “Do you think you might try smoking cigarettes in the future?”, with the following response options: probably yes, probably not, or definitely not. The following brief measure developed by our group was used to assess the girls’ stages of change (ie, maintenance, action, preparation, contemplation, and precontemplation) related to avoidance of SHS: “When you are exposed to secondhand cigarette smoke, do you consistently do things to reduce your exposure to the smoke?”, with the following response options: yes, I have been for more than 6 months; yes, I have been, but for less than 6 months; no, but I intend to in the next 30 days; no, but I intend to in the next 6 months; or no, and I do not intend to in the next 6 months [35].

### Statistical Analysis

The primary aim of the analysis was to determine whether differences in the outcomes were associated with exposure to the intervention versus control messages. To account for possible confounding not controlled by random allocation, potential confounders were identified with bivariate tests—any variables found to differ (*P*<.10) between the treatment and control groups were included as covariates in subsequent multivariate models. Bivariate analyses of the categorical data were conducted with Fisher’s exact tests. Follow-up time (measured in months) was included in the multivariate models. A generalized estimating equation (GEE) was used for all multivariate regression models to adjust the standard errors of the parameter estimates for the correlated responses of students clustered within the same school [36]. Adjusted relative risks (ARRs) were estimated using a modified Poisson regression, with robust error variance [37] originally proposed by Lee and Chia [38] for binary outcomes [39]. The robust error variance estimator was used because Poisson regressions overestimate the standard errors of parameters arising from binary outcomes [37,40]. All statistical analyses were conducted with IBM SPSS Statistics Version 19.0. Although not part of the original aim, supplementary stratified analyses were performed to evaluate the impact of the intervention among the group of girls who requested more information about the relationship between cigarette smoke and (breast) cancer, and then among girls who did not request more information.

## Results

### Characteristics

A total of 745 nonsmoking adolescent girls completed the baseline survey, and 618 completed both the baseline and follow-up surveys, providing a retention rate of 83.0%. Among the 127 girls lost to follow-up, 59 (46.5%) and 68 (53.5%) were in the control and intervention groups, respectively—results from a one-sample binomial test (hypothesized proportions of 50% in intervention and control groups) indicated that the difference in proportions lost to follow-up was not statistically significant (*P*=.48). Therefore, 618 nonsmoking girls from 74 schools (some students reported moving to a new school which increased the number of schools from 48 to 74), aged 13 to 15 years (mean age of 14 years), were included in the analyses. Of these girls, 242 received the intervention message and 376 received the standard control message. Among girls in the intervention group, 55 out of 242 (22.7%) requested more information about the relationship between cigarette smoke and breast cancer, and 64 girls out of 376 (17.0%) in the control group requested more information about the relationship between cigarette smoke and cancer—these proportions did not significantly differ (*P*=.52) by group. On average, 6 months elapsed between the baseline presentation of the messages and the follow-up assessment. Table 1 provides the participants’ characteristics as well as the SHS exposure characteristics. Differences in age at follow-up, family income at baseline, family history of breast cancer at follow-up, and parental smoking status at follow-up were statistically significant (*P*<.10) between intervention and control groups.

**Table 1:** Personal and secondhand smoke exposure characteristics of nonsmoking adolescent girls by group allocation.

Characteristic			Control, n (%) (n=376)	Intervention, n (%) (n=242)	Total, n (%) (n=618)	*P* value^a^
**Demographics**						
	**Age in years (at follow-up)**					
		13	20/376 (5.3)	5/242 (2.1)	25/618 (4.0)	<.001
		14	157/376 (41.8)	56/242 (23.1)	213/618 (34.5)	
		15	199/376 (52.9)	181/242 (74.8)	380/618 (61.5)	
	**Ethnicity (at baseline)**					
		Aboriginal	27/372 (7.3)	22/237 (9.3)	49/605 (8.1)	.50
		Non-Aboriginal	343/370 (92.7)	215/237 (90.7)	558/608 (91.8)	
	**Family income (at baseline)**					
		Below average	24/348 (6.9)	7/233 (3.0)	31/574 (5.4)	.04
		Average	280/348 (80.5)	183/231 (79.2)	463/579 (80.0)	
		Above average	44/349 (12.6)	41/231 (17.7)	85/578 (14.7)	
	Responded “Yes” to “Family history of breast cancer” (at baseline)		66/357 (18.5)	64/236 (27.1)	130/591 (22.0)	.02
**SHS exposure**						
	Responded “Yes” to “Parent(s) smoke(s)” (at follow-up)		101/338 (29.9)	40/223 (17.9)	141/562 (25.1)	.001
	Responded “Yes” to “Friends smoke” (at follow-up)		57/295 (19.3)	27/191 (14.1)	84/488 (17.2)	.18
	Answered “Yes” to “Does anyone smoke in your home every day or almost every day?” (at follow-up)		41/363 (11.3)	17/239 (7.1)	58/604 (9.6)	.12
	**Past month’s exposure to SHS (at follow-up)**					
		Every day	16/372 (4.3)	5/238 (2.1)	21/600 (3.5)	.60
		Almost every day	38/368 (10.1)	27/237 (11.4)	65/607 (10.7)	
		At least once a week	103/369 (27.9)	68/236 (28.8)	171/604 (28.3)	
		At least once in past month	166/369 (45.0)	111/236 (47.0)	227/495 (45.8)	
		Never	46/368 (12.5)	25/236 (10.6)	71/607 (11.7)	
**Other characteristics**						
	**Intentions to try smoking in future (at baseline)**					
		Probably yes	16/363 (4.4)	5/238 (2.1)	24/585 (4.1)	.71
		Probably not	73/361 (20.2)	47/238 (19.7)	102/586 (17.4)	
		Definitely not	272/361 (75.3)	185/238 (77.7)	459/585 (78.5)	
	Time elapsed to follow-up, months (SD)		5.82 (0.77)	5.84 (1.10)	5.83 (0.94)	.77^b^

^a^Based on Fischer’s exact test.

^b^Based on independent samples *t* test.

### Knowledge and Risk Perceptions of Cigarette Smoke Exposure and Breast Cancer at Follow-Up

As shown in Table 2, after adjusting for differences in age, family income, family history of breast cancer, parental smoking status, and time elapsed to follow-up, the girls who received the intervention message were 33% more likely than girls that received the control message to identify breast cancer as an illness caused by exposure to cigarette smoke (ARR 1.33, 95% CI 1.05-1.68).

After adjusting for differences in age, family income, family history of breast cancer, parental smoking status, and time elapsed to follow-up, the girls who received the intervention message were 10% more likely than girls in the control group to agree with the statement that being exposed to SHS increases their risk of breast cancer (ARR 1.10, 95% CI 1.02-1.20) (see Table 2).

**Table 2:** Knowledge, risk perceptions, smoking behavior, intentions, and stage of change related to cigarette smoke exposure and breast cancer, by group allocation, at follow-up.

Follow-up assessment			Control, n (%) (n=376)	Intervention, n (%) (n=242)	Total, n (%) (n=618)	ARR^a^ or unadjustedRR^b^ (95% CI)
**Knowledge and risk perceptions**						
	Responded “Yes” to “Breast cancer is caused by exposure to cigarette smoke.”		107/376 (28.5)	96/242 (39.7)	203/618 (32.8)	1.33^a^ (1.05-1.68)
	Responded “Agree” to “Being exposed to secondhand cigarette smoke increases my risk of getting breast cancer.”^c^		252/314(80.3)	199/224 (88.8)	451/538 (83.8)	1.10^a^ (1.02-1.20)
**Smoking behavior and intentions**						
	Responded “Yes” to “Tried smoking.”^d^		13/376 (3.5)	9/239 (3.8)	22/613 (3.6)	1.14^b^ (0.48-2.69)
	**Intentions to try smoking in the future**					
		Probably yes or probably not	75/354 (21.2)	51/231 (22.1)	126/585 (21.5)	1.00^a^ (0.98-1.03)
		Definitely not	279/354 (78.8)	180/231 (77.9)	459/585 (78.5)	
**Stage of change related to avoidance of SHS**						
	Answered “Yes” to “When you are exposed to secondhand cigarette smoke do you consistently do things to reduce your exposure to the smoke?”^e^		241/296 (81.4)	161/196 (82.1)	402/492 (81.7)	0.97^a^ (0.82-1.15)

^a^Adjusted relative risk for differences in income, age, parental smoking status, family history of breast cancer, and time elapsed to follow-up.

^b^Unadjusted relative risk (URR) for differences in income, age, parental smoking status, family history of breast cancer, and time elapsed to follow-up.

^c^“Strongly agree” and “agree” were collapsed as “agree”, and “strongly disagree” and “disagree” were collapsed as “disagree”, which was the reference group.

^d^Either tried cigarettes or roll-your-own cigarettes in the time between baseline and follow-up.

^e^Responded either “Yes, for more than 6 months” or “Yes, but for less than 6 months.”

### Smoking Behavior, Intentions to Smoke, and Stage of Change Related to Avoidance of SHS

As shown in Table 2, no statistically significant treatment effects were observed for a change in smoking status (ie, having tried cigarette smoking between baseline and follow-up), intentions to try smoking, or stage of change related to avoidance of SHS.

### Stratified Analyses to Investigate Impact of Intervention Within Groups Defined by Requesting More Information

After stratifying groups by whether or not the girls requested more information about the relationship between cigarette smoke and (breast) cancer, we examined all outcomes analyzed in the prior multivariate models (knowledge and risk perceptions, smoking behavior and intentions, and stage of change related to avoidance of SHS) using univariate analyses (Pearson Chi-square tests). Among girls who requested more information (n=119), a significantly greater proportion of girls in the intervention group (27/48, 56%) compared with the control group (14/71, 20%) identified breast cancer as an illness caused by exposure to cigarette smoke (*P*=.003). Among girls who did not request more information (n=499), a significantly greater proportion of girls in the intervention group (170/194, 87.6%) compared with the control group (244/305, 80.0%) agreed with the statement that being exposed to SHS increases their risk of breast cancer (*P*=.03). All other outcomes of interest did not significantly differ in these stratified analyses.

## Discussion

### Principal Findings

The objectives of this study were to evaluate the effects of a targeted gender- and Aboriginal status-sensitive, breast cancer-specific, Web-based message on nonsmoking adolescent girls’ (1) knowledge of the causal link between cigarette smoke exposure and breast cancer, (2) perceptions of breast cancer risk associated with exposure to cigarette smoke, (3) smoking initiation, (4) intentions to smoke, and (5) stage of change related to avoidance of SHS at follow-up (ie, 6 months after the message delivery). The results indicate that the intervention message had positive effects on awareness of cigarette smoke exposure as a causal agent of, and risk factor for, breast cancer among nonsmoking girls approximately 6 months following message dissemination. Compared with the girls who received the standard control message, the girls who received the intervention message were 33% more likely to identify breast cancer as an illness caused by exposure to cigarette smoke and 10% more likely to agree with the statement that being exposed to SHS increases their risk of breast cancer. Furthermore, a similar pattern of results was found in groups stratified by whether or not the girls requested more information about the relationship between cigarette smoke and (breast) cancer.

This is the first intervention we are aware of specifically designed to evaluate the effect of a brief Web-based intervention on awareness of the relationship between SHS and breast cancer. Furthermore, this study considered both changes in perceived risk regarding the link between exposure to SHS and breast cancer, as well as smoking intentions and behavior at follow-up. Two teams of researchers have tested online interventions to reduce cigarette use and intentions to smoke among adolescents [26,28]. They both reported reduced odds of future smoking intentions, and the analyses by Norman et al revealed reduced odds of cigarette use by the nonsmokers in the intervention arm. However, these studies utilized multicomponent interventions. Norman et al implemented a 5-phase, interactive program in Smoking Zine [26], which included a virtual point-of-sale that evaluated the cost of smoking, followed by smoking use assessments and a pros versus cons evaluation of smoking and being smoke free. Buller et al evaluated Consider This, a comprehensive 6-module program based on Bandura’s Social Cognitive Theory, which included assessments of perceived social norms concerning the prevalence of smoking, future smoking expectations, and smoking prevalence among adolescents (resistance efficacy) [28]. In comparison, our intervention was brief, which may explain why we found significant gains in knowledge, but no change in actual behavior.

Findings from our study, which indicate that brief, targeted, breast cancer-specific messages can raise girls’ awareness of the link between breast cancer and cigarette smoke exposure, add to the small but growing body of literature about the benefits of using the Internet to deliver messages to raise awareness and ultimately effect health behavior change. For example, in a recent study 25% of 497 adolescents reported changing their behavior (eg, nutrition and/or physical activity) based on findings in online searches for health information [24]. Indeed, several trials have demonstrated that Web-based health promotion interventions can be targeted and widely disseminated in an effective and relatively inexpensive manner [41,42]. In addition, there may be the possibility of using existing online commercial marketing services to disseminate targeted messages on a large scale at very low cost. Although the results of this study indicate that the START messages would likely increase awareness of the risk of breast cancer associated with cigarette smoke exposure, further research is needed to determine how changes in awareness or perceived risk could be leveraged to include subsequent reductions in smoking initiation and SHS exposure.

### Limitations

This study is not without limitations. The purpose was to compare a generic control message with targeted gender- and Aboriginal status-sensitive, breast cancer-specific (intervention) messages. Given that the control message did not contain breast cancer-specific information it is not possible to disentangle the influence of the breast cancer-specific information from the other aspects of the gender tailoring of the intervention message. The positive outcomes of the intervention were increases in girls’ knowledge and risk perceptions related to cigarette smoke exposure and breast cancer, not with actual behavior change (eg, reduced smoking initiation or stage of change related to avoidance of SHS). However, the latter, nonsignificant findings could have resulted from the small number (22/613) of girls who reported having tried smoking at the time of follow-up. Future studies could address this limitation by employing a much larger sample. These studies could add to the results of the current investigation by examining behavioral change among an oversampling of girls’ who have already tried smoking. Additionally, if a second no-information control group had been included as a reference (ie, one that did not receive any message), larger intervention effects may have been found. The generalizability of these findings is limited—they may not be relevant to other age groups and ethnicities.

### Conclusions

The study findings suggest that a brief, targeted, disease-specific and gender-sensitive, Web-based message influenced girls’ knowledge of cigarette smoke exposure as a risk factor for, and causal agent of, breast cancer, thereby supporting the use of this type of intervention in future trials. Brief informational interventions delivered via the Internet appear to be effective, far-reaching forms of health promotion that have the potential for long-term effects on adolescents’ knowledge and risk perceptions with regard to cigarette exposure and breast cancer. Future investigations of Web-based interventions could employ repeated exposure of the targeted message, or they could implement a multistep design and incorporate short message service (SMS) text messaging or interactive voice responses to understand how to increase the effectiveness of these types of interventions.

## Abbreviations

ARR: adjusted relative risk
BASUS: British Columbia Adolescent Substance Use Survey
GEE: generalized estimating equation
SHS: secondhand smoke
SMS: short message service
START: Supporting Tailored Approaches to Reducing Tobacco
URR: unadjusted relative risk

## References

[1] Boffetta P, Autier P (2011). Is breast cancer associated with tobacco smoking?. BMJ.

[2] Johnson KC, Miller AB, Collishaw NE, Palmer JR, Hammond SK, Salmon AG, Cantor KP, Miller MD, Boyd NF, Millar J, Turcotte F (2011). Active smoking and secondhand smoke increase breast cancer risk: the report of the Canadian Expert Panel on Tobacco Smoke and Breast Cancer Risk (2009). Tob Control.

[3] Luo J, Margolis KL, Wactawski-Wende J, Horn K, Messina C, Stefanick ML, Tindle HA, Tong E, Rohan TE (2011). Association of active and passive smoking with risk of breast cancer among postmenopausal women: a prospective cohort study. BMJ.

[4] Morabia A (2002). Smoking (active and passive) and breast cancer: epidemiologic evidence up to June 2001. Environ Mol Mutagen.

[5] Nishioka T, Kim HS, Luo LY, Huang Y, Guo J, Chen CY (2011). Sensitization of epithelial growth factor receptors by nicotine exposure to promote breast cancer cell growth. Breast Cancer Res.

[6] Sadri G, Mahjub H (2007). Passive or active smoking, which is more relevant to breast cancer. Saudi Med J.

[7] Xue F, Willett WC, Rosner BA, Hankinson SE, Michels KB (2011). Cigarette smoking and the incidence of breast cancer. Arch Intern Med.

[8] Collishaw NE, Boyd NF, Cantor KP, Hammond SK, Johnson KC, Millar J, Miller AB, Miller M, Palmer JR, Salmon AG, Turcotte F (2009). Canadian Expert Panel on Tobacco Smoke and Breast Cancer Risk.

[9] Bjerkaas E, Parajuli R, Weiderpass E, Engeland A, Maskarinec G, Selmer R, Gram IT (2013). Smoking duration before first childbirth: an emerging risk factor for breast cancer? Results from 302,865 Norwegian women. Cancer Causes Control.

[10] Ha M, Mabuchi K, Sigurdson AJ, Freedman DM, Linet MS, Doody MM, Hauptmann M (2007). Smoking cigarettes before first childbirth and risk of breast cancer. Am J Epidemiol.

[11] Reynolds P, Goldberg D, Hurley S, Nelson DO, Largent J, Henderson KD, Bernstein L (2009). Passive smoking and risk of breast cancer in the California teachers study. Cancer Epidemiol Biomarkers Prev.

[12] (2012). Health Canada.

[13] Leatherdale ST, Ahmed R (2009). Second-hand smoke exposure in homes and in cars among Canadian youth: current prevalence, beliefs about exposure, and changes between 2004 and 2006. Cancer Causes Control.

[14] Haines RJ, Bottorff JL, Barclay McKeown S, Ptolemy E, Carey J, Sullivan K (2010). Breast cancer messaging for younger women: gender, femininity, and risk. Qual Health Res.

[15] McBride CM, Emmons KM, Lipkus IM (2003). Understanding the potential of teachable moments: the case of smoking cessation. Health Educ Res.

[16] Slovic P (2000). What does it mean to know a cumulative risk? Adolescents' perceptions of short-term and long-term consequences of smoking. J Behav Decis Mak.

[17] Kong G, Singh N, Krishnan-Sarin S (2012). A review of culturally targeted/tailored tobacco prevention and cessation interventions for minority adolescents. Nicotine Tob Res.

[18] Krebs P, Prochaska JO, Rossi JS (2010). A meta-analysis of computer-tailored interventions for health behavior change. Prev Med.

[19] Noar SM, Benac CN, Harris MS (2007). Does tailoring matter? Meta-analytic review of tailored print health behavior change interventions. Psychol Bull.

[20] Noar SM, Grant Harrington N, Van Stee SK, Shemanski Aldrich R (2011). Tailored health communication to change lifestyle behaviors. Am J Lifestyle Med.

[21] Skinner CS, Campbell MK, Rimer BK, Curry S, Prochaska JO (1999). How effective is tailored print communication?. Ann Behav Med.

[22] Strecher VJ (1999). Computer-tailored smoking cessation materials: a review and discussion. Patient Educ Couns.

[23] Bottorff JL, Haines-Saah R, Oliffe JL, Struik LL, Bissell L, Gotay C, Hutchinson P, Richardson C, Johnson K (2014). Designing tailored messages about smoking and breast cancer: a focus group study with youth. Canadian J Nursing Res.

[24] Ettel G, Nathanson I, Ettel D, Wilson C, Meola P (2012). How do adolescents access health information? And do they ask their physicians?. Perm J.

[25] Vance K, Howe W, Dellavalle RP (2009). Social Internet sites as a source of public health information. Dermatol Clin.

[26] Norman CD, Maley O, Li X, Skinner HA (2008). Using the Internet to assist smoking prevention and cessation in schools: a randomized, controlled trial. Health Psychol.

[27] Strecher VJ, McClure JB, Alexander GL, Chakraborty B, Nair VN, Konkel JM, Greene SM, Collins LM, Carlier CC, Wiese CJ, Little RJ, Pomerleau CS, Pomerleau OF (2008). Web-based smoking-cessation programs: results of a randomized trial. Am J Prev Med.

[28] Buller DB, Borland R, Woodall WG, Hall JR, Hines JM, Burris-Woodall P, Cutter GR, Miller C, Balmford J, Starling R, Ax B, Saba L (2008). Randomized trials on consider this, a tailored, Internet-delivered smoking prevention program for adolescents. Health Educ Behav.

[29] Patten CA, Croghan IT, Meis TM, Decker PA, Pingree S, Colligan RC, Dornelas EA, Offord KP, Boberg EW, Baumberger RK, Hurt RD, Gustafson DH (2006). Randomized clinical trial of an Internet-based versus brief office intervention for adolescent smoking cessation. Patient Educ Couns.

[30] Woodruff SI, Conway TL, Edwards CC, Elliott SP, Crittenden J (2007). Evaluation of an Internet virtual world chat room for adolescent smoking cessation. Addict Behav.

[31] Prokhorov AV, Kelder SH, Shegog R, Murray N, Peters R, Agurcia-Parker C, Cinciripini PM, de Moor C, Conroy JL, Hudmon KS, Ford KH, Marani S (2008). Impact of A Smoking Prevention Interactive Experience (ASPIRE), an interactive, multimedia smoking prevention and cessation curriculum for culturally diverse high-school students. Nicotine Tob Res.

[32] Rice VH, Weglicki LS, Templin T, Jamil H, Hammad A (2010). Intervention effects on tobacco use in Arab and non-Arab American adolescents. Addict Behav.

[33] Richardson CG, Struik LL, Johnson KC, Ratner PA, Gotay C, Memetovic J, Okoli CT, Bottorff JL (2013). Initial impact of tailored Web-based messages about cigarette smoke and breast cancer risk on boys' and girls' risk perceptions and information seeking: randomized controlled trial. JMIR Res Protoc.

[34] (2011). Health Canada.

[35] Richardson CG, Schwartz J, Struik LL, Bottorff JL (2013). Adapting the Stage of Change model to investigate adolescent behavior related to reducing second hand smoke exposure. Open J Prev Med.

[36] Fitzmaurice GM (2011). Applied Longitudinal Analysis, 2nd edition.

[37] Zou G (2004). A modified poisson regression approach to prospective studies with binary data. Am J Epidemiol.

[38] Lee J, Chia KS (1993). Estimation of prevalence rate ratios for cross sectional data: an example in occupational epidemiology. Br J Ind Med.

[39] Barros AJ, Hirakata VN (2003). Alternatives for logistic regression in cross-sectional studies: an empirical comparison of models that directly estimate the prevalence ratio. BMC Med Res Methodol.

[40] Zocchetti C, Pesatori AC, Bertazzi PA (2004). A simple method for risk assessment and its application to 1,3-butadiene. Med Lav.

[41] Cugelman B, Thelwall M, Dawes P (2011). Online interventions for social marketing health behavior change campaigns: a meta-analysis of psychological architectures and adherence factors. J Med Internet Res.

[42] Mermelstein R, Turner L (2006). Web-based support as an adjunct to group-based smoking cessation for adolescents. Nicotine Tob Res.

